# Microbiota Composition and Functional Profiling Throughout the Gastrointestinal Tract of Commercial Weaning Piglets

**DOI:** 10.3390/microorganisms7090343

**Published:** 2019-09-12

**Authors:** Raphaële Gresse, Frédérique Chaucheyras Durand, Lysiane Dunière, Stéphanie Blanquet-Diot, Evelyne Forano

**Affiliations:** 1 Université Clermont Auvergne, UMR 454 MEDIS UCA-INRA, F-63000 Clermont-Ferrand, France; stephanie.blanquet@uca.fr (S.B.-D.); evelyne.forano@inra.fr (E.F.); 2 Lallemand SAS, 19 rue des Briquetiers, BP59, F-31702 Blagnac CEDEX, France; fchaucheyrasdurand@lallemand.com (F.C.D.); lduniere@lallemand.com (L.D.)

**Keywords:** weaning, piglets, microbiota, gastrointestinal tract

## Abstract

Dietary, environmental, and social stresses induced by weaning transition in pig production are associated with alterations of gut microbiota, diarrhea, and enteric infections. With the boom of -omic technologies, numerous studies have investigated the dynamics of fecal bacterial communities of piglets throughout weaning but much less research has been focused on the composition and functional properties of microbial communities inhabiting other gastrointestinal segments. The objective of the present study was to bring additional information about the piglet bacterial and archaeal microbiota throughout the entire digestive tract, both at the structural level by using quantitative PCR and high-throughput sequencing, and on functionality by measurement of short-chain fatty acids and predictions using Tax4Fun tool. Our results highlighted strong structural and functional differences between microbial communities inhabiting the fore and the lower gut as well as a quantitatively important archaeal community in the hindgut. The presence of opportunistic pathogens was also noticed throughout the entire digestive tract and could trigger infection emergence. Understanding the role of the intestinal piglet microbiota at weaning could provide further information about the etiology of post-weaning infections and lead to the development of effective preventive solutions.

## 1. Introduction

In intensive swine production, early weaned piglets encounter social, environmental and dietary stresses generated by handling, transport, mixing litters, separation from the mother and the transition from a milk-based diet to a solid-based diet [1]. Dietary changes at weaning are associated with low feed and water intake, gastrointestinal (GI) changes and modifications of the intestinal piglet microbiota [2]. This huge microbial community has numerous roles benefiting the host including protection from pathogenic bacteria [3,4] and its alteration has already been linked with numerous diseases or infections [5]. Weaning transition in the piglet is associated with diarrhea and enteric infections which are the main causes of piglet death [6] and could be a direct consequence of microbial shifts observed at this critical period. Besides raising an economic burden in pig industry, weaning associated infections rear public health concerns due to the massive use of antimicrobials for therapeutic purposes [2,7]. 

Thus, there is a crucial need to gain further knowledge about weaning piglet microbiota functionality and composition to define non-pharmacological strategies to counteract post-weaning diarrhea. With the development of omics technologies, numerous of studies have characterized the fecal microbiota composition of weaning piglets and its response towards weaning transition, antibiotic use, dietary changes, presence of pathogens or feed additives [8,9,10,11,12,13,14]. However, microbial interactions, activities and fermentations also occur in all the other segments of the pig gastrointestinal tract (GIT) which possess different physicochemical environments and, in consequence, different microbial communities [15]. Additionally, the small and large intestine are preferably used as colonization or infection sites by opportunistic pathogens and should be more largely studied to unravel the mechanisms inducing dysbiosis and infections. Only a few studies describe the microbial populations longitudinally harbored in the weaning piglet GIT using next-generation sequencing techniques and revealed distinct location-specific differences in microbial composition inside the different gut compartments [16,17,18,19,20]. Among these studies, four out of five used animals raised in a controlled laboratory environment [16,17,18]. However, as microbial population colonizing the intestine is impacted by the environment, including the use of commercial pigs in such research is also of great interest to be representative of swine industry conditions. 

The microbial communities inhabiting the digestive tract have numerous functions such as participating to the digestion of organic compounds and fermentation of carbohydrates to make metabolites accessible for the host [3,4]. The differences of microbiota distribution throughout the entire GIT of piglets suggest that microbiota from different intestinal niches may have different roles [21]. Functions of weaning piglets GIT microbiota is yet to be explored. New bioinformatic tools such as PICRUSt [22] or Tax4Fun [23] were developed in the last decade and should be of a great help to predict the functionality of microbiome. Only one previous study investigated the functional predictions of piglet GIT microbiota, two weeks after weaning [21]. Functional capacities varied according to gut locations and from mucosal to luminal samples [21]. However, analyses of microbiome functions throughout the diverse GIT segments of weaning piglets remains to be performed. At least, if all piglet gut microbiome studies focus on bacterial composition, no omics investigations involve the archaeal microbiota yet.

The objective of the present study was to provide more information about the bacterial and archaeal GIT microbiota composition and functions of six male Landrace x Large White commercial piglets using 16S rDNA sequencing with Illumina MiSeq platform, quantitative PCR, gas chromatography for short-chain fatty acids (SCFA) quantification and the Tax4Fun pipeline. Sampling was performed on the commercial weaning day at 28 days of age on digesta from the stomach, duodenum/jejunum, ileum, cecum, proximal colon, distal colon, and feces. Mucosal scrapings from proximal colon, the most documented fermentative organ, were also collected to compare weaning piglet microbiota population and functions from the lumen to the mucosa. 

## 2. Materials and Methods

### 2.1. Animal and Sample Collections

All experimental procedures were followed in accordance with the C2E2A Local Ethic Committee and the guidelines established by the European Community Concil under the Directive 2010/63/EU. The experiments were exempted from ethic evaluations because all animals were commercially raised and slaughtered on site under the supervision of the local veterinary. Animals were raised in a conventional pig farm located in the Haute-Loire area of the Auvergne-Rhône-Alpes region in France. All piglets remained with their mother and siblings during the suckling period. None of the selected piglets received antibiotic or had signs of enteric or metabolic disturbances from birth until slaughter. In addition to sow milk, piglets received water and pre-weaning diet (Supplementary Table S1) ad libitum. Six healthy male piglets, originating from a different mother, weighting between 10 and 12 kilos, (Landrace × Large White) at 28 days old, corresponding to the day of weaning, were randomly picked among the litters and slaughtered in order to collect their GIT. Immediately postmortem, the entire digestive tract was removed. The entire stomach; duodenum/jejunum, first part after the stomach’s pyloric sphincter and the middle part of the small intestine; ileum, last part of the small intestine before the ileocecal valve; cecum; proximal colon; first part of the large intestine after the ileocecal valve; distal colon, last part of the large intestine just before the rectum and rectum segments were carefully doubled clamped to avoid digesta leakage, kept at 4 °C and quickly transferred to the laboratory. Once reaching the laboratory facility, the GIT segments were separated by cutting between the double clamp of each section. Each segment of the digestive tract was weighted. pH was measured directly inside the organs using a pH1970 I pH meter (Wissenschaftlich-Technische Werkstätten GmbH, Weilheim, Germany) coupled with a LoT 406-M6-DKK-S7/25 probe (Mettler Toledo, Columbus, OH, USA). The pH probe was carefully rinsed with water after each pH measurement, and calibrated between each animal. The luminal contents of each intestinal compartment were entirely removed, pooled, mixed, aliquoted and immediately stored at −80 °C. The organs were washed using sterile 0.9% phosphate-buffer saline (PBS) and weighted again. For mucosal scrapings, the proximal colon was rinsed with sterile PBS to remove any digesta and its surface was scrapped using a sterile 76 × 25 mm Menzel-Gläser Superfrost (Thermo Scientific, Waltham, MA, USA) microscope slide. The scrapings were then stored at −80 °C. 

The cecal, colonic and fecal concentrations of SCFAs were quantified by gas chromatography to determine the concentrations and proportions of acetate, propionate, isobutyrate, butyrate, caproate, isovalerate, and valerate. Approximately 500 μL of digestive contents were weighted, suspended with 500 μL of sterile water, mixed and centrifuged (14,000 g, 10 min, 4 °C). Eight hundred microliters of cell free supernatants were mixed with 500 μL of 0.4% (*w/v*) crotonic acid and 2% (*w/v*) metaphosphoric acid solutions. This mixture was centrifuged again, and the supernatant obtained was injected in a PerkinElmer Clarus 580 gas chromatograph (Waltham, Massachusetts, USA) for quantification of SCFA. The Mann and Whitney U test was used to compare the concentration of the main SCFA between GIT segments using the RStudio software version 1.0 (with R statistical package version 3.3.1, R Development Core Team, http://www.R-project.org).

### 2.2. DNA Extraction from Digestive Contents and Colon Mucosal Scrapings

Total DNA was extracted using the Quick-DNA Fecal/Soil Microbe Miniprep Kit (Zymo Research, Irvine, CA, USA) according to the manufacturer’s instructions. The quality of the eluted DNA was assessed by agarose gel electrophoresis. Extracts were quantified using the Qubit dsDNA Broad Range Assay Kit (Invitrogen, Carlsbad, CA, USA) with a Qubit 2.0 Fluorometer (Invitrogen, Carlsbad, CA, USA). Samples were stored at −20 °C prior to use.

### 2.3. Quantification of Bacteria and Methanogenic Archaea Populations by QPCR

Two specific primer pairs were used for quantitative PCR (qPCR). The total bacteria were quantified using16SrDNA primers 338F 5’- ACTCCTACGGGAGGCAG-3’ [24] and 530R 5’- GTATTACCGCGGCTGCTG-3’ [25]. Methanogenic archaea were targeted using 16SrDNA primers 1174F 5’- GAG GAAGGAGTGGACGACGGTA-3’ and 1389R 5’- ACGGGCGGTGTGTGCAAG-3’ [26]. Real-time PCR assays were performed on a Rotor-Gene Q (Qiagen, Venlo, NL) in 20 µL reactions with QuantiFast SYBR GREEN master mix (Qiagen, Venlo, NL) with the additions of each primer at a concentration of 0.5 µM. The total bacteria 16S rDNA gene and 16S rDNA Archaea gene were respectively amplified using the following program: 2 min denaturation at 95 °C and 10 min denaturation at 95 °C; 40 and 45 cycles of 20 s at 95 °C and 60 s elongation and extension at 61 °C; and a melt curve step from 60 °C to 95 °C.

### 2.4. Standard Curve Assessments for Quantitative PCR Analyses

Conventional PCR for the amplification of the 16S ribosomal gene was carried out on genomic DNA from *Escherichia coli* (DSMZ N° 30083) and *Methanobrevibacter smithii* (DSMZ N° 861) (DSMZ, Braunschweig, Germany). The reaction was performed using the universal 16S primers 8F 5’-ACTCCTACGGGAGGCAG-3’ and 1492R 5’-GTATTACCGCGGCTGCTG-3’ and the Platinum™ *Taq* DNA Polymerase kit (Invitrogen, Carlsbad, CA, USA). The PCR was carried out with a Bio-Rad iCycler thermal cycler (Bio-Rad, Hercules, CA, USA) under the following conditions: one cycle of 94 °C for 2 min; 30 cycles of 94 °C for 30 s, 52 °C for 30 s, and 72 °C for 90 s. The PCR products were purified using the QIAquick PCR Purification kit (Qiagen, Venlo, NL) according to the manufacturer’s instructions and were subjected before and after purification to a 1% agarose gel electrophoresis containing ethidium bromide and visualized for being approximately equal to 1484 bp using the ladder 500 bp Mol Ruler (Bio-Rad, Hercules, CA, USA). DNA concentration was measured via the Qubit dsDNA Broad Range Assay Kit (Invitrogen) with a Qubit 2.0 Fluorometer (Invitrogen, Carlsbad, CA, USA). The 16S rDNA gene copy number was calculated using the formula: copy number/μL= (C/X)*0.912.10^12^ with C: DNA concentration measured (ng/μL) and X: PCR fragment length (bp/copy) and diluted in 10-fold dilution series to be used as qPCR standards.

### 2.5. MiSeq 16S RDNA Sequencing and Bioinformatic Analysis

Prior to PCR amplification, the DNA concentration of all samples was measured using the Qubit dsDNA High Sensitivity Assay Kit (Invitrogen, Carlsbad, CA, USA) with a Qubit 2.0 Fluorometer (Invitrogen, Carlsbad, CA, USA) and diluted to 2 ng/µL. The Bacterial V3-V4 region of 16S rDNA and the Archaeal 16S rDNA were respectively amplified with primers 357F 5′-CCTACGGGNGGCWGCAG-3’ and 805R 5′-GACTACHVGGGTATCTAATCC-3’ and primers 349F 5′-GYGCASCAGKCGMGAAW-3’ and 806R 5′-GGACTACVSGGGTATCTAAT -3’. Amplicons were generated using a Fluidigm Access Array followed by high-throughput sequencing on an Illumina MiSeq system (Illumina, San Diego, CA, USA) performed at the Carver Biotechnology Center of the University of Illinois (Urbana, IL, USA). The demultiplexed paired end Illumina sequence reads in the FastQ format were uploaded into the Galaxy instance (v.2.3.0) of the Genotoul bioinformatics platform (http://sigenae-workbench.toulouse.inra.fr) to be used in the FROGS (Find Rapidly OTU with Galaxy Solution) pipeline [27]. During the FROGS pre-process, sequences were depleted of barcode and the sequences with a non-appropriate length or containing ambiguous bases were removed. Next, reads were clustered into de novo operational taxonomic units (OTUs) using SWARM algorithm [28] with, at first, a denoising step to build very fine cluster using the minimal distance equal to 1 and, secondly, with an aggregation distance equal to 3. Chimeras were then detected and removed with VSEARCH [29]. Additionally, filters were applied to the OTUs in order to remove singletons [30,31]. The OTUs selected were taxonomically assigned using the Silva release 132 reference database [32]. 

### 2.6. Statistical Analysis of Sequencing Data 

Statistical analysis was processed using the RStudio software version 1.0 (with R statistical package version 3.5.1, R Development Core Team, http://www.R-project.org). OTU structure and composition analyses were performed using the phyloseq R package [33]. Visualization of data was performed using the ggplot2 R package. Alpha diversity indices were calculated using a paired non-parametric t-test with the following indices: Inverse Simpson index, Chao 1 index, number of observed OTU phylogenetic diversity (PD) and Shannon index. Prior to beta diversity calculations, rarefaction using the transform counts methods was applied to the dataset. Statistical differences in Bray Curtis distance between GI locations were tested using a multi-analysis of variance (MANOVA) performed with ADONIS using the vegan R package [34] with 9999 permutations and represented by principal coordinate analysis (PCoA) plots. A non-metric multi-dimensional scaling (NMDS) on Bray Curtis distance was employed to visualize the differential abundance of main bacterial genera across the GIT segments using heatmap representations. Statistical comparisons of mucosa versus digesta samples from proximal colon were performed using the Wald test of the DESeq2 R package at the genus level. 

### 2.7. Function Prediction with Tax4Fun

Tax4Fun, an online tool hosted into Galaxy (v.2.3.0) of the Genotoul bioinformatics platform (http://sigenae-workbench.toulouse.inra.fr), was used to predict functional genes of prokaryotic microorganisms across GIT sections [23]. Tax4Fun transforms OTUs picked up against the Silva 123 database into a taxonomic profile of KEGG (Kyoto Encyclopedia of Genes and Genomes) organisms and normalize these predictions by the 16S rDNA copy number. The most important functions of each intestinal segment were graphically represented using the Rstudio software version 1.0 (with R statistical package version 3.5.1, R Development Core Team, http://www.R-project.org).

## 3. Results

### 3.1. Physiological Parameters of the GIT of Piglets

The weight of organs and pH data collected while sampling is represented in Figure S1. As expected, the pH measured in the stomach was lower than that of the other anatomical regions of the GIT. Individual variability was remarkably reduced for the pH data of the cecum and proximal colon contents (Figure S1A). Segments from the foregut displayed higher weight but also greater inter-individual variability compared to hindgut anatomical regions (Figure S1B). 

### 3.2. Concentration and Proportion of SCFAs Detected in the Hindgut

The SCFA concentrations varied in the different anatomical regions of GIT and was also quite variable among individuals. However, the total SCFA concentrations reached the highest values in the cecum followed by the proximal colon. Distal colon and rectum contents shared approximately the same total concentration of SCFAs. Acetic, propionic and butyric acids were the major SCFAs found in all compartments (Table 1). The concentrations of acetic acid were significantly higher in the cecum compared to proximal colon (*p*-value = 0.025) and in the proximal colon compared to distal colon (*p*-value = 0.008), but were not different between the distal colon and rectum contents (*p*-value > 0.05). Butyric and propionic acids were detected in significantly higher concentration in proximal colon compared to distal colon (*p*-value = 0.015). The relative abundance of acetate over total SCFAs tended to be lower in the proximal colon and very similar in the other segments, from 62.1 to 63.9% (Figure S2). The relative abundance of propionic acid was the highest in proximal colon and the lowest in rectum content. The relative abundance of butyric acid slightly increased from the cecum to the distal colon from 9.4% to 11%, with an intermediate proportion observed in the rectum.

### 3.3. QPCR Quantification of Total Bacteria and Methanogenic Archaea

**Figure 1:**
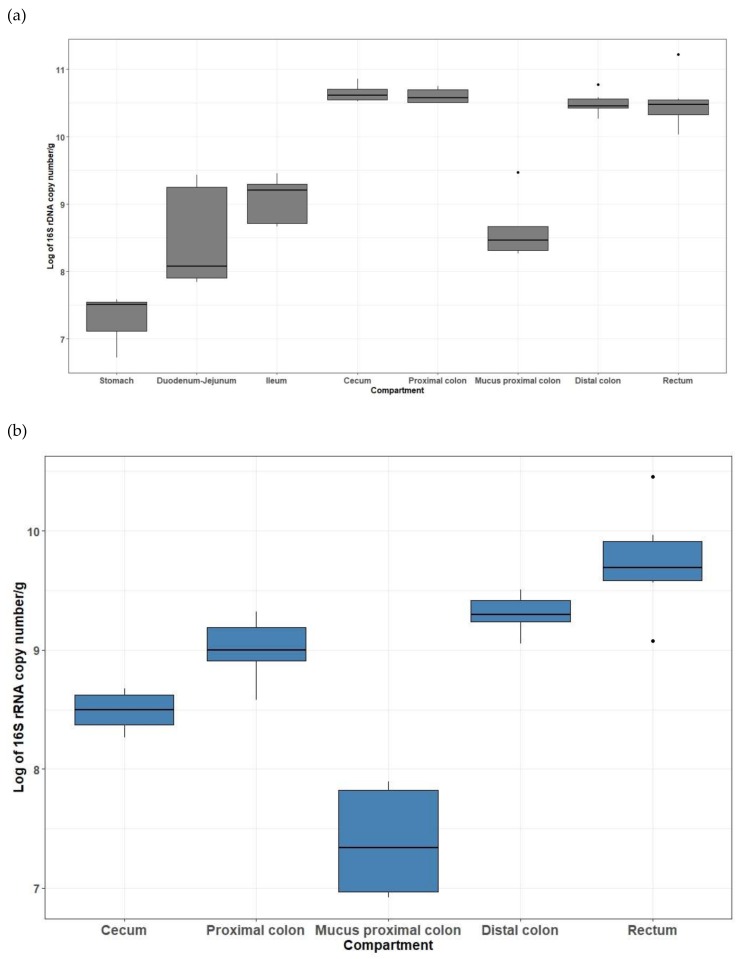
Quantification of total bacteria (**a**) and archaea (**b**) populations along the GIT of weaning piglet using qPCR on the 16S rRNA gene.

Total bacteria were quantified in higher concentrations in the large intestine than in the stomach and small intestine regions, and were less variable between individuals in the lower parts of the gut (Figure 1A). The means of the log of total bacteria 16S copy numbers were increasing from the stomach (7.3 log_10_ copies/g) to the ileum (9.1 log_10_ copies/g), and were close to 10.5 in the lower gut segments. In the mucus collected in the proximal colon, the mean concentration (16S copy number) was 9.0 log_10_ copies/g. Methanogen archaea were not detected in samples from stomach and small intestine, but were quantified with an increasing gradient in digesta from the cecum (8.5 log_10_ copies/g) to the rectum (9.7 log_10_ copies/g) (Figure 1B). They were also detected in the mucus of the proximal colon, but in lower concentration (7.4 log_10_ copies/g). 

### 3.4. MiSeq Sequencing Data

A total number of 48 digestive contents and mucosal samples were collected during June to July 2017. The Illumina MiSeq run generated a total of 18,358,526 high quality sequences, lowered to 17,121,898 after removal of PhiX control reads. Primer sorted metrics total numbers of reads were equal to 5,364,263 and 1,882,030 respectively for V3-V4 16S primer set and the archaea primer set. Chimera removal, quality filtering and deletion of singletons led to a total of 12,970 identified taxa. The initial mean (± standard deviation) number of sequences per sample was 19,744 (± 6755) for the V3-V4 run and 15,467 (± 3086) for the cecum, proximal, and distal colon and rectum samples of the archaea run. After removal of chimeras and singletons, the mean (± standard deviation) number of sequences per sample was finally 12,361 (± 6374) for the V3-V4 run and 13,895 (± 2756) for the cecum, proximal, and distal colon and rectum samples of the archaea run. For the archaeal primer set, the mucosal samples collected at the proximal colon level displayed a lower mean (± standard deviation) number of sequences per sample equal to 2084 (± 1487) and 1643 (± 1384) after removal of chimeras and singletons. The very low number of sequences (<350 per samples) confirmed a poor detection of archaea in the upper part of piglet GIT.

### 3.5. Bacterial Communities All Along the GIT

#### 3.5.1. At the Phylum Level

**Figure 2:**
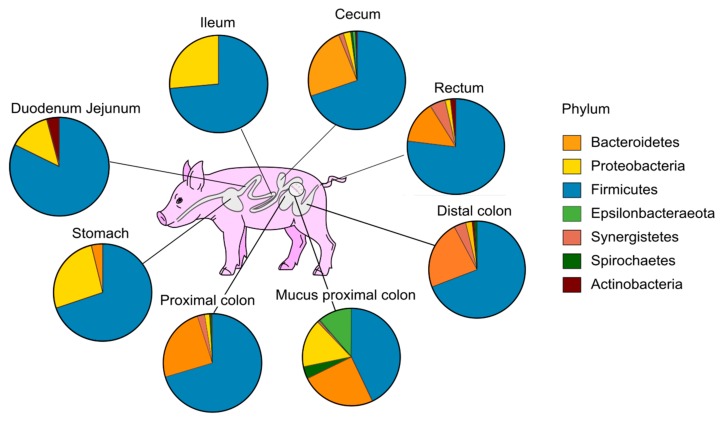
Mean relative abundance of the phylum-level microbiota across the weaning piglet GIT.

The most abundant phyla Firmicutes, Proteobacteria and Bacteroidetes displayed distinct profiles along the piglet GIT (Figure 2). The bacterial communities found in the duodenum and jejunum belonged predominantly to the Firmicutes phylum. The Proteobacteria phylum appeared particularly established in the gastric and ileal compartments, reaching ~ 25% of mean relative abundance. However, these upper GIT regions were submitted to high inter-individual variability (Figure S3). Despite a slight variability of the Firmicutes: Bacteroidetes ratio, the lower gut exhibited more diverse but more similar profiles between individuals (Figure 2 and Figure S3). Overall, the major phylum displayed in the lower gut segments was the Firmicutes followed by the Bacteroidetes, this latter being present with an increased proportion in colon areas compared with rectum content (Figure 2). The Proteobacteria and Epsilonbacteraeota phyla were better represented in the mucus samples. Supplementary Figure S3 also highlighted several particularities such as the presence of the phylum Synergistetes in one individual all along the lower part of the gut. 

#### 3.5.2. At the Lower Taxonomic Level

**Figure 3:**
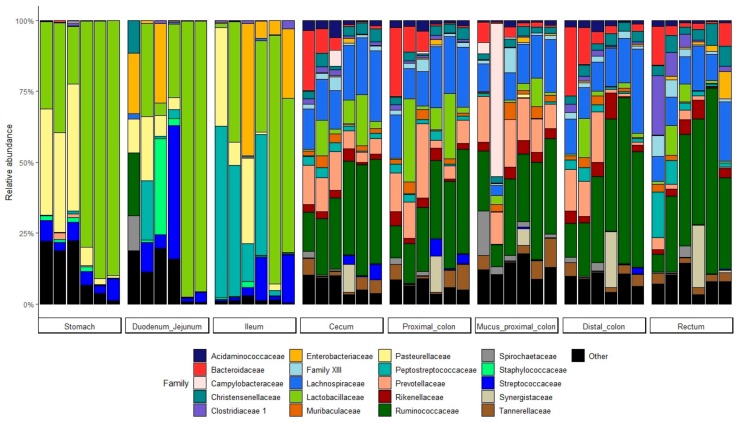
Relative abundance of the main bacterial families in the stomach and intestine segments of 6 weaning piglets.

**Figure 4:**
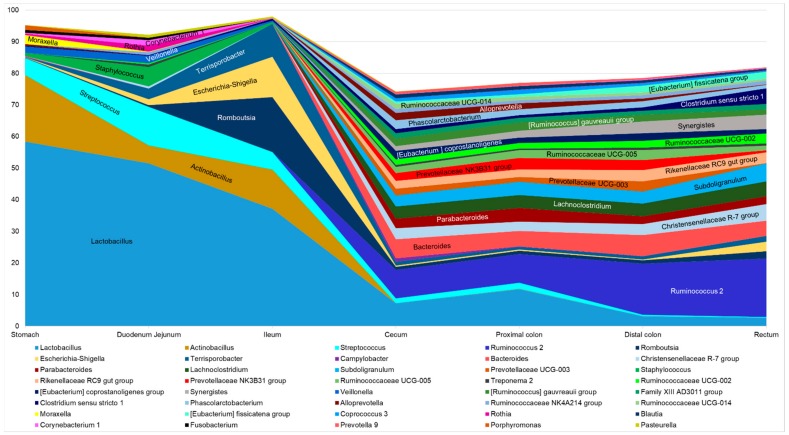
Mean relative abundance of the main bacterial genera along the GIT of weaning piglets.

The microbiota in the stomach and small intestine was inter-individually variable and principally composed of *Lactobacillaceae* and *Pasteurellaceae* families in the stomach, in addition to *Peptostreptococcaceae* and *Streptococcaceae* families in the small intestine (Figure 3). The *Enterobacteriaceae* family was also present in the small intestine, mainly in the ileum. The main families harbored in the hindgut were the *Ruminococcaceae*, *Lachnospiraceae*, *Prevotellaceae*, and the *Bacteroidaceae*. In the lower part of the gut, the variability across individuals was reduced. However, in the rectum, a reduced proportion of *Prevotellaceae* and *Bacteroidiaceae* was observed and members of *Enterobacteriaceae*, *Peptostreptococcaceae* or *Clostridiaceae* were found in variable abundances between individuals (Figure 3). The overall biolocalization of piglet bacterial community composition at the genus level is presented in Figure 4. The highest diversity was found in cecum, colon, and rectum segments which contain a great number of genera mainly consistent across samples. Many genera showed great specificity towards their location throughout the gut (Figure 4). For example, *Ruminococcaceae* and *Prevotellaceae* genera, *Rikenellaceae* RC9 gut group, *Lachnoclostridium*, *Parabacteroides*, and *Treponema* seemed to colonize almost exclusively the hindgut. Although *Staphylococcus* was mainly identified in the upper parts of the gut, no genus displayed absolute specificity toward the stomach or small intestine samples. (Figure S4) Some genera, such as *Campylobacter*, were not consistently present across individuals (Figure S4). 

### 3.6. Archaeal Microbiota Throughout the GIT

**Figure 5:**
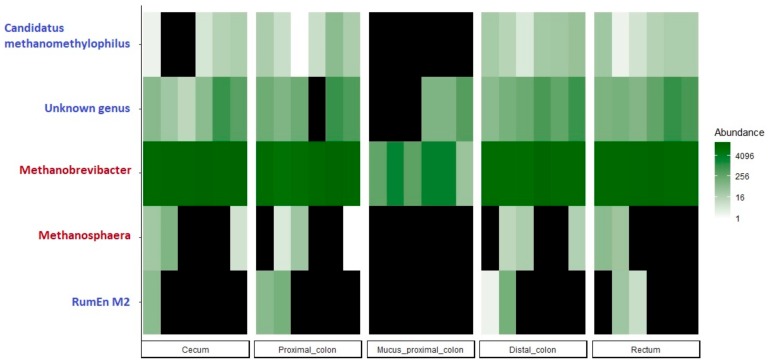
Top 20 of the most abundant archaeal OTUs along the GIT of weaning piglets (blue names correspond to *Methanomethylophilaceae* family and red names correspond to *Methanobacteriaceae* family, black color corresponds to an abundance of 0).

Archaeal community was exclusively composed of members belonging to the *Euryarchaeota* phylum (Figure S5). Despite some inter-individual variability across samples, the most represented genus was *Methanobrevibacter* belonging to the *Methanobacteriaceae* family reaching 98.1% of total sequences. Few other identified taxa were members of the *Methanomethylophilaceae* family belonging to the *Methanomassiliicoccales* order and representing 1.8% of total sequences (Figure 5). 

### 3.7. Diversity and Richness Throughout the GIT Segments 

#### 3.7.1. Alpha Diversity and Species Richness

**Figure 6:**
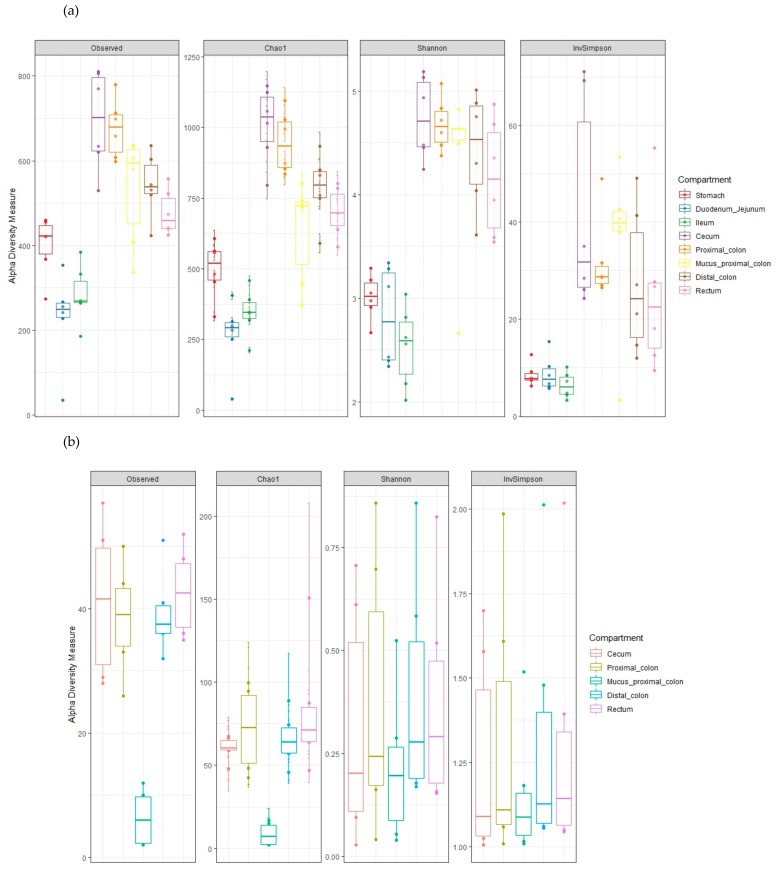
Alpha diversity measures on bacterial (**a**) and archaeal (**b**) OTUs across the GIT of weaning piglets.

To further explore the microbiota structure among the different GI segments, bacterial and archaeal alpha diversity was evaluated according to the observed OTUs and the Shannon, Chao1 and inverse Simpson indices (Figure 6). Bacterial species richness and evenness, determined by the Shannon index, increased considerably from the small intestine to the hindgut (Figure 6A). The observed OTUs, chao1 index and inverse Simpson indices, reflecting respectively, the richness of species, the number of taxa observed in a sample and the species number and their abundance, displayed a similar profile confirming the increase of alpha diversity toward the cecum, colon, and rectum segments. Regarding the archaea, the hindgut digestive contents displayed similar alpha diversity indices (Figure 6B). For the mucosal colon samples, for both bacteria, and archaea, the observed OTUs and Shannon indices were numerically variable and lower than those found in the corresponding proximal colon lumen samples (Figure 6A,B).

#### 3.7.2. Microbial Community Analysis

**Figure 7:**
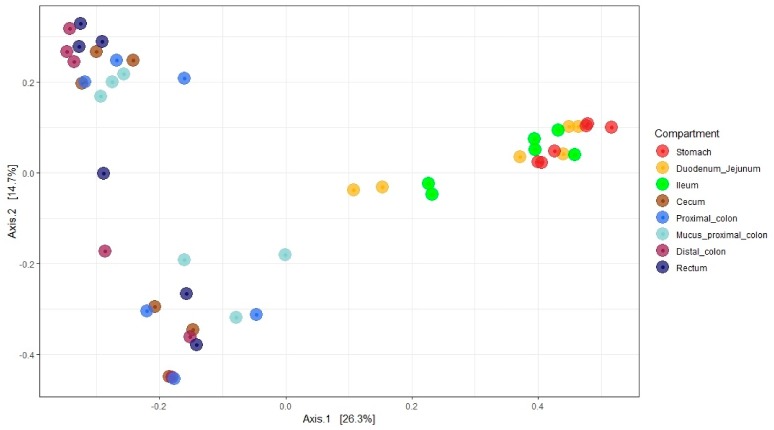
Bray Curtis multi-dimensional scaling / principal coordinate analysis of the bacterial communities across weaning piglet GI organs.

Principal coordinate analysis was used to determine the similarities of microbial communities between different GI locations. Multi-dimensional scaling (MDS)/ principal coordinate analysis (PCoA) on Bray Curtis distance showed that stomach and small intestine samples formed a distinct cluster and could be separated from the samples collected in the hindgut. The mucus proximal samples could not be differentiated from the lumen proximal colon samples on the PCoA plot. Overall, the communities clustered by lower and upper position in the GIT (ADONIS: Bray Curtis, *P*-value = 1e-04, *R*-value = 0.266) (Figure 7).

### 3.8. Differential Analysis of Mucosal Versus Luminal Proximal Colon Samples

**Figure 8:**
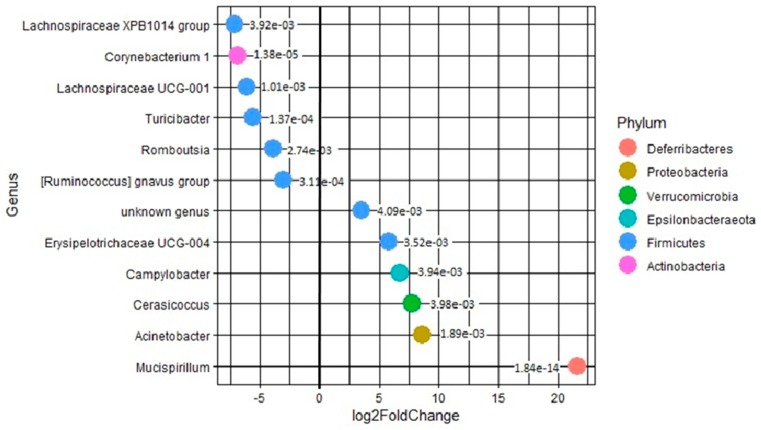
Differentially abundant genera between proximal colon mucosal scrapping and proximal colon digesta. Only the statistically significant genera are represented (*p* values are indicated on the figure).

Following the Wald test performed using the DESEQ2 R package, several OTUs were identified as significantly more abundant in mucosal scrapings compared with luminal digestive content (Figure 8). Among them, the genus *Mucispirillum* was found to be the most prevalent in mucus from proximal colon compared with luminal colonic content with a Log2FoldChange > 20, followed by the *Acinetobacter* and *Cerasicoccus* genera (7 > Log2FoldChange > 9) (Figure 8).

### 3.9. Prediction of Microbiota Functional Capacity

**Figure 9:**
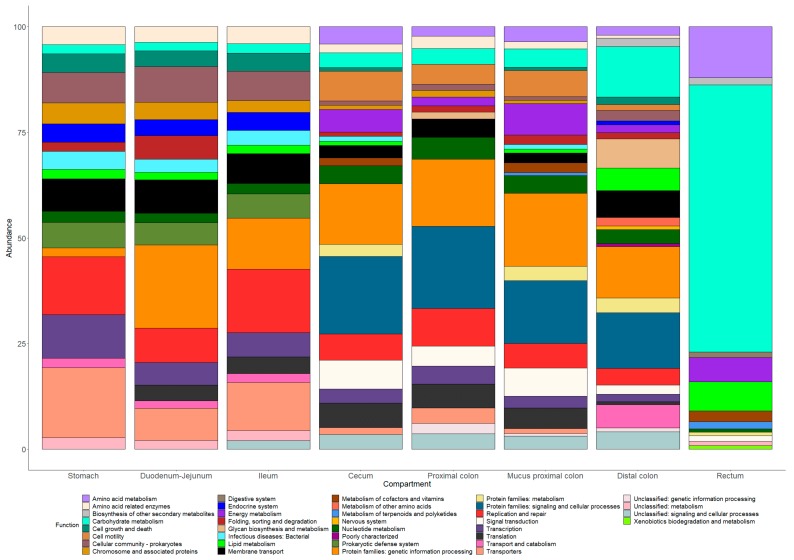
Prediction of functional capacity of the bacterial communities along the GIT of weaning piglets.

Functional profiles were obtained through the Tax4Fun tools (Abhauer2015). Like for the microbiota composition, the stomach, duodenum, jejunum, and ileum displayed similar predicted functional profiles. In these compartments, the major identified pathways were DNA replication and repair, transport, genetic information processing and cellular community (Figure 9). Some functions were mostly predicted in these parts of the GIT such as infectious disease, prokaryotic defense system and endocrine system (Figure 9). The cecum and proximal colon (lumen and mucus) harbored very close profiles but rather different from those predicted in the upper part segments. Metabolic information related to signaling and cellular processes, signal transduction, energy metabolism, amino acid metabolism, and cell motility appear to be expanded in these areas. The metabolism of terpenoids and polyketides appears to be a specificity of the proximal colon mucus samples (Figure 9). In the distal colon segment, the relative abundances of several metabolic pathways increased compared to other colonic segments such as transport and catabolism, glycan synthesis, and carbohydrate metabolism. At last, the rectum content demonstrated a very distinctive profile. The functional capacity predicted in this area was mainly focused on carbohydrate, amino acids, lipid and energy metabolism and xenobiotics biodegradation and metabolism (Figure 9). 

## 4. Discussion

Studies focusing on the weaning period raise special interests for swine production due to the high potential health concerns resulting from the increase of bacterial infections and post-weaning diarrhea. This study contributes to the growing knowledge of piglet microbiota structure and functions by combining compositional and structural sequencing and quantitative data. In this field, the -omics techniques considerably facilitated microbiome-oriented research in the past decade. Most of the metagenomic investigations in recent years described the shifts induced by weaning on piglet fecal bacterial communities [8,9,10,11,12,13,14]. However, the main feature reported in the present study was a clear separation between the upper part and the lower part of the piglet GIT, suggesting that characterization of fecal microbiota could be inadequate to investigate post-weaning infections etiology due to the fact numbers of pathogen infection or multiplication sites are located in the jejunum, ileum, or the colon segments. The segregation between the small and large intestine compartments was visible from the reported weight of organs to the microbial numbers and diversity, as assessed by qPCR and high-throughput sequencing. This could be due to shifts in the physicochemical conditions and differences of substrate availability between the superior and inferior gut segments as suggested elsewhere [15,17,35]. In our study, samples from small intestine were the most subjected to inter-individual variability in terms of composition but also bacterial diversity as reported in weaning piglets [19] but also in other animals [36,37]. The lack of stability of foregut bacterial communities could be due to the discontinuous influx of food depending on feed intake and diet type as a pre-weaning diet was offered to piglets in addition to sow milk. Indeed, several individuals harbored a great majority of *Lactobacillaceae* suggesting a preference for suckling behavior. It is also important to mention that the lower quantity of total bacteria detected in small intestine segments could impact the stability of bacterial composition. Genetics could also play a role in shaping the piglet intestine microbiota in this study as all animals were raised in different litters but in the same environment with the same diet at their disposal.

In the present study, the dominant phylum was attributed to the *Firmicutes* all along the GIT. Similar findings were described in fecal samples of 25- to 28-day-old weaning piglets and in all the GIT compartment of 42 day old weaned piglets from a close cross breed (Duroc × Landrace × Large White) [7,11,20]. The *Firmicutes* were also reported to be the most dominant in jejunum, ileum and colon of 28 day old German Large White x Piétrain weaning piglets [16]. Interestingly, the *Bacteroidetes* phylum was found to be the most represented in cecum content of 24 day old weaned piglets from the same cross breed as used in the present study (Landrace × Large White), suggesting a possible role of rearing environment or diet [38]. Despite the highest prevalence of *Firmicutes* across all the samples of our study, members of the *Bacteroidetes* phylum were found in higher relative abundance in cecum and colon mucosal scrapings and digestive contents. Across all GIT sites, OTUs classified as the most prevalent were *Lactobacillus, Actinobacillus, Romboutsia, Escherichia-Shigella, Terripsorobacter, and Campylobacter.* Up to now, the genus *Actinobacillus* has never been referenced as one of the most abundant in weaning piglet intestinal microbiota. Nonetheless, this taxon was identified to be predominant in piglet oropharynx which could explain its high abundance in stomach and small intestine samples of our study [39]. The *Lactobacillus* genus was already found to be predominant in the fecal microbiota of two weeks old suckling piglets [12]. In our study, the *Lactobacillus* genus was present in all the samples starting from 60% of relative abundance in stomach digesta and decreasing across the digestive tract to reach less than 5% of relative abundance in rectum content, suggesting the real dynamic of piglet microbiota across GIT segments. *Terripsorobacter* and *Romboutsia* were also detected throughout the whole GIT segments in our samples. These two genera belonging to *Clostridium* cluster XI, were previously characterized as dominant in the intestine of piglets regardless of their age [19]. Several species belonging to these genera are considered to be anaerobic pathogens [19,40,41].

*Escherichia-Shigella* species are facultative aerobes which are usually detected in higher proportion close to the intestinal mucosa due to the higher oxygen concentration released by the epithelial barrier in this area [17]. Even though members of this group cannot be distinguished by Illumina sequencing techniques, the *Escherichia-Shigella* genus also hosts a variety of opportunistic pathogens such as enterotoxigenic *E. coli* (ETEC). ETEC is a very common cause of post-weaning diarrhea in piglets and is known to act mainly in the small intestine [12]. In our study, OTUs belonging to *Escherichia-Shigella* genus were detected in higher proportion in duodenum/jejunum and ileum samples, consistently with the potential site of action of ETEC strains. Additionally, *Escherichia-Shigella* group was detected in higher relative abundance in mucosal scrapings sampled from porcine colon confirming the affinity of this group for mucus secreting enterocytes [42]. A study from Bin et al. revealed that ETEC-induced diarrhea led to changes in the microbiota composition in the jejunum and feces of 18 day old piglets [6]. At genus level, diarrheal piglets had an increased percentage of *Lactococcus* in jejunum microbiota and *Escherichia* in feces, a lower abundance of *Prevotella* in feces and a lower abundance of *Escherichia* in jejunum [6]. Interestingly, piglets that recovered from diarrhea harbored a higher percentage of *Escherichia* genus in their jejunal microbiota suggesting that microbiota may play a resistant role to diarrhea after exposure to inducers [6]. The *Campylobacter* OTUs were also mainly found in mucosal scrapings of proximal colon, especially in one particular animal which was however devoid of intestinal lesions. *Campylobacter* species are known to adhere to the mucus surface, produce toxins and activate inflammation, leading to a reduction in nutrient use efficiency [19]. However, in pig gut, *Campylobacter* is considered to be a commensal bacterium [19,43]. *Campylobacter jejuni* and *Campylobacter coli* were even identified as the two most prevalent species in pigs at various ages [19,43]. A cecum mucus layer enriched in *Campylobacter* has been already observed, which supports the results of the present study, though it concerned only some individuals [44]. Other differences between mucosa and lumen were observed in our work using DESEQ2 analysis. The genus *Mucispirillum* belongs to the *Deferribacteres* phylum and is represented by a single species *Mucispirillum schaedleri,* which has a strong predilection for mucosal surfaces [13,45]. The latter information is consistent with our findings, as the *Mucispirillum* was 20 times more abundant in mucosal versus luminal proximal colon samples. *Mucispirillum* members were already detected in an increased amount in helminth infected pigs [46,47] but also in post-weaning piglet feces [11,13]. The *Acinetobacter* genus was also identified in a higher relative abundance in mucosal samples. *Acinetobacter* are strict anaerobes which were previously detected in high proportion in milk-fed piglet colon and were positively correlated with a metabolic pathways involved in the invasion of intestinal epithelial cells [48]. Interestingly, these bacteria are known to have heterotrophic nitrification and aerobic denitrification capabilities [49]. These data highlight the fact that gut compartments of healthy weaning piglets shelter a reservoir of opportunistic pathogens which could take advantage of any stress or dysbiosis related to weaning and thereby trigger pathologies. 

This study contributes to increase our knowledge about the composition and the diversity of archaeal piglet microbiota all along the GIT. No data were previously published about the archaeal microbiota of piglets all along the GIT. Archaea are a separate domain of life inhabiting the GIT of animals initially studied regarding the high levels of methane initiated in livestock [50]. Methanogens diversity in piglets is yet poorly understood. One metagenomic study performed in growing pig mid colon revealed that archaeal species are influenced by diet composition [51]. Additionally, the unique study performed using PCR-DGGE analysis on the fecal archaeal microbiota of weaning piglets revealed a shift from the *Methanobrevibacter boviskoreani* to *M. smithii* species during weaning transition [52]. Our findings revealed that despite their poor diversity, archaea seem to be present in relatively high amounts in the hindgut, reaching 10^8^ to 10^10^ 16S gene copy numbers per gram of digestive content from cecum to feces, where ecological conditions allow their establishment. All along the intestine, the most represented archaeal group was the genus *Methanobrevibacter* followed by an unknown genus from the *Methanomethylophilaceae* family belonging to the *Methanobrevibacter* order and *Thermoplasmata* class, both present in every sample from the hindgut including mucosal scraps of proximal colon. The genus *Methanobrevibacter* was already previously reported to be predominant in pig feces [52,53,54]. Su et al. 2014 [55] indicated the species *Methanobrevibacter smithii* was the most represented species in pre-weaning piglet fecal samples replaced by *M. boviskoreani* post-weaning. The genus *Candidatus methanomethylophilus* displayed a lower number of counts in our samples but seems to be present in almost every segment of the luminal hindgut. The *Methanomassiliicoccales* order has been already identified in diverse anaerobic environments including the GIT of humans and animals [56], but to our knowledge, this is the first time this group is detected in pigs. 

Very few data are available about functional properties of bacterial and archaeal GI microbiota of weaning piglet. This is important to increase our understanding of the potential contribution of microbiota to the physiology and metabolism of young piglets. Microbial fermentation products such as SCFAs represent the major source of carbon from non-digestible carbohydrates to the host and play numerous roles in host metabolic health [57]. Changes of SCFA luminal concentration and composition were previously reported in the large intestine of piglets after weaning [58]. In this study, the three main SCFAs: butyrate, acetate, and propionate, were detected in higher concentrations in cecum and proximal colon contents compared to distal colon and rectum samples. Indeed, the process of SCFA absorption remains not clear in weaning piglets, only 5% of the produced SCFAs in the hindgut is thought to be generally excreted in feces [58,59]. A study from Nakatani et al. (2018) [58] evaluated the SCFA concentrations in the cecum of 28 day old piglets and found similar concentration of butyrate and propionate, respectively 10 and 15 mmol/kg. However, the concentration of acetate described by Nakatani et al. was approximatively twice lower than the one detected in cecal samples of this study. This difference could be attributed to variabilities in piglet microbiota composition between the two studies. Acetate production is yet widely distributed among bacteria and cannot be related to specific bacterial groups [57]. The *Ruminococcaceae, Clostridiaceae* and *Lachnospiraceae* families known to produce butyric acid [60] were identified in our study with a higher relative abundance inside cecum and proximal colon segments consistently with the higher concentration of butyric acid detected in these compartments. Propionic acid is mainly provided by *Prevotellaceae*, *Bacteroidiaceae* families or the *Negativicutes* class [59]. In our samples, the lower concentration of propionate in distal colon and rectum digesta was in concordance with the low relative abundance of *Prevotellaceae, Bacteroidiaceae, Acidaminococcaceae, and Phascolarctobacterium.* Isovaleric acid has been previously positively correlated with *Christensenella* and *Methanobrevibacter* genera in human microbiota [61]. In our study, isovalerate shown to be twice more present in rectum content compared to other hindgut segments. Interestingly, our qPCR and metagenomic results highlighted a higher quantity of methanogenic archaea and a higher relative abundance of *Christensenellaceae* group in the rectum area. 

The methanogenic archaeal community represent microbiota keystone species by transforming end-product from bacterial fermentation such as hydrogen, carbon dioxide or acetate into methane and potentially influencing the overall gut microbial populations [50,51]. In feces, the *Methanobrevibacter* genera performs hydrogenotrophic methanogenesis and transforms hydrogen and carbon dioxide derived from bacterial fermentations into methane and H_2_O [52]. The use of H_2_ and CO_2_ as an energy source is thought to optimize fermentation and oxidation processes [62]. Like in other animal GIT, *Methanomassiliicoccales* probably carry out methylotrophic methanogenesis in the pig intestine [56]. These data suggest the main function of the archaeal microbiota of piglet would be CH_4_ production and could potentially play an important role in piglet health.

With the recent rise of –omic technologies, the KEGG pathways obtained using Tax4Fun analysis offers the possibility to predict putative functions carried out by gut bacteria. In piglets, such analyses were already performed in fecal samples by Hu et al. (2016) [11] and Dou et al. (2017) [63] using the commonly used PICRUST tool, which is comparable to Tax4Fun in terms of robustness [64], but never all along the entire GIT as performed in our study. Within our sequencing results, two distinct types of profiles were generated by functional predictions in the piglet GIT segments. In stomach, duodenum/jejunum and ileum contents, most of the identified pathways belonged to microbial physiology such as DNA replication and repair, genetic information processing, membrane transport or pathways related to cellular community which might be explained by the adaptation of microorganisms to a challenging physicochemical environment. Also, in weaning piglets, gut organs are still maturing which probably induces a very tight microbe-host crosstalk and the expression of numerous pathways related to prokaryotic cellular machinery. In the cecum, proximal colon, proximal colon mucosal scrapings, and distal colon, pathways related to energy, amino acids and carbohydrate metabolism were detected in higher abundance compared to foregut compartments probably due to the higher abundance of *Clostridium, Lachnospiraceae, and Prevotelleaceae* members. However, the most represented pathways in piglet hindgut belonged to prokaryotic signaling and cellular processes which may highlight the high communication level between microorganisms, their competition for persisting inside the same ecological niches and the progressive establishment of the still immature microbial intestinal ecosystem of weaning piglets. The metabolic pathways involving the metabolism of terpenoids and polyketides was only detected in proximal colon mucosal scrapings. Terpenoids and polyketides are large families of active natural compounds produced by a wide variety of living form including bacteria [65,66] suggesting that the emergence of this pathway in our mucosal samples could be due to differences in the relative abundances of other metabolic routes. In the distal colon segments, the relative abundance of pathways belonging to amino acid, carbohydrate, lipid and glycan metabolism and biosynthesis of secondary metabolites is increased compared to cecum and proximal colon area potentially due to lower level of host-microbiota crosstalk in this area. The rectum segments harbored a totally different profile. Indeed, contrary to the other GIT segments, no pathway was related to cellular processes or host-microbiota crosstalk. In the rectal area, carbohydrate metabolism was the most abundant followed by amino acid metabolism, glycan biosynthesis, fatty acid biosynthesis, and metabolism of cofactors and vitamins, consistent with the study of Hu et al. 2016 [11] focusing exclusively on piglet fecal samples. The distinct metabolic profile of rectum contents could be explained by an absence of, or much reduced exchanges between the microbiota and host epithelial cells. Even though the KEGG pathways obtained via Tax4Fun analysis stays predictions which must be considered carefully, the present results strongly emphasize the need for investigations about the activity of microbiota all along the small and large intestine of weaning piglets.

## 5. Conclusions

To conclude our findings, participate to the gain of knowledge about the composition and possible functional properties of microbial population inhabiting the total digestive tract of healthy commercial weaning piglets. Our study highlighted the strong differences in the shaping of weaning piglet maturing microbiota between GIT segments and the need to further explore the functional microbiota living inside the small and large intestines. Two of the main observations made throughout this study was the detection of an abundant archaeal microbiome in the large intestine as well as the presence of opportunistic pathogens inside the fore and lower piglet gut before weaning. This pathogen reservoir may trigger infection emergence during the critical weaning period leading to severe post-weaning diarrhea and massive antibiotic use. Therefore, understanding the role of the intestinal microbiota in preventing or eliciting the emergence of pathogens during the sensible weaning period in commercial piglets is of a great importance to find effective preventive actions to reduce the risk of post-weaning infections. 

## Figures and Tables

**Table 1:** Concentrations in mmol/L of SCFA measured in hindgut compartments of 28-day-old piglets by gas chromatography. (std= standard deviation).

	Concentration of Short-Chain Fatty Acid (mmol/L)															
Compartments	Acetate		Butyrate		Propionate		Valerate		Caproate		Iso Butyrate		Iso Valerate		Total SCFAs	
	Individual Values	Mean ± std	Individual Values	Mean ± std	Individual Values	Mean ± std	Individual Values	Mean ± std	Individual Values	Mean ± std	Individual Values	Mean ± std	Individual Values	Mean ± std	Individual Values	Mean ± std
**Caecum**	67.41	60.87 ± 15.75	8.03	9.06 ± 2.55	13.28	17.67 ± 4.68	3.12	3.17 ± 0.85	0.40	0.37 ± 0.17	2.28	2.28 ± 0.63	2.57	2.84 ± 0.73	97.09	96.24 ± 22.15
	54.52		8.68		17.61		2.93		0.06		2.53		3.58		89.92	
	78.89		10.11		15.83		3.82		0.49		2.89		3.13		115.17	
	33.90		4.72		13.18		1.63		0.34		1.09		1.50		56.36	
	71.05		10.96		25.15		3.58		0.35		2.66		3.26		117.00	
	59.43		11.84		20.95		3.92		0.55		2.23		2.99		101.91	
**Proximal colon**	33.18	39.00 ± 12.70	6.23	7.09 ± 2.37	12.88	14.03 ± 5.99	1.77	2.18 ± 0.83	0.08	0.23 ± 0.12	1.47	1.44 ± 0.55	2.59	2.23 ± 0.48	58.20	66.20 ± 21.59
	47.73		7.10		10.78		2.31		0.37		1.87		2.40		72.55	
	27.37		4.74		8.92		1.15		0.10		0.89		1.68		44.84	
	46.17		7.34		20.87		1.99		0.33		1.33		2.26		80.29	
	55.72		11.52		22.11		3.64		0.26		2.25		2.82		98.32	
	23.84		5.63		8.60		2.19		0.23		0.85		1.63		42.98	
**Distal colon**	12.61	17.46 ± 6.65	1.17	3.11 ± 2.21	3.75	5.03 ± 3.34	0.23	0.89 ± 0.83	0.00	0.11 ± 0.12	0.44	0.46 ± 0.18	1.06	1.05 ± 0.44	19.28	28.11 ± 12.86
	11.97		1.26		1.70		0.31		0.03		0.30		0.58		16.14	
	13.06		1.43		2.34		0.23		0.00		0.20		0.54		17.80	
	29.10		4.30		10.82		1.14		0.26		0.62		1.32		47.57	
	17.01		3.84		5.10		1.09		0.13		0.50		1.16		28.83	
	21.01		6.63		6.48		2.35		0.24		0.68		1.67		39.05	
**Rectum**	17.41	21.23 ± 7.47	4.51	3.51 ± 2.12	6.72	4.34 ± 2.60	0.56	1.03 ± 0.68	0.00	0.19 ± 0.21	1.25	0.90 ± 0.59	2.92	2.03 ± 1.18	33.37	33.22 ± 12.33
	9.41		1.83		1.49		0.56		0.10		0.28		0.70		14.37	
	29.08		1.13		5.27		0.64		0.00		0.52		1.26		37.90	
	29.18		6.69		7.71		2.22		0.53		1.88		3.87		52.07	
	21.50		4.64		2.88		1.49		0.17		0.89		2.04		33.60	
	20.82		2.24		1.96		0.72		0.31		0.56		1.40		28.02	

## Supplementary Materials

The following are available online at https://www.mdpi.com/2076-2607/7/9/343/s1, Figure S1: Physiological data on the GIT of weaning piglets: (a) pH values inside different locations of the GIT, (b): Weight of each segment of the intestine with (blue) or without the total content (red), Figure S2: Mean of relative abundance of SCFAs in cecum. proximal colon. distal colon and rectum of weaning piglets, Figure S3: Relative abundance of the main bacterial phyla in the stomach and intestine segments of 6 weaning piglets, Figure S4: Top 20 of the most abundant bacterial OTUs along the GIT of weaning piglets. OTUs’ colors correspond to phyla assignation (Dark green = *Spirochaetes*. yellow = *Proteobacteria*. blue = *Firmicutes*. light green = *Epsilonbacteraeota*. orange = *Bacteroidetes*, black: no detection), Figure S5: Relative abundance of the main archaeal families in the low intestine segments of 6 weaning piglets, Table S1: Description of the commercial pre-weaning diet. The table contains the analytical composition of a pelleted diet that remains at the disposal of piglet from the second week of the nursing period. Ingredients: barley. wheat. extruded wheat. whey powder. soybean meal. wheat flour. oat flakes. protein isolates from whey powder. colza oil. soy protein isolate. potato protein. milk powder. vegetable oils. sugar.
