# Supplementary material for: Microbiota Composition and Functional Profiling Throughout the Gastrointestinal Tract of Commercial Weaning Piglets
Source: Microorganisms. 2019 Sep 12;7(9):343. doi: 10.3390/microorganisms7090343 (PMC6780805; doi:10.3390/microorganisms7090343)
Supplement: Supplementary file 1 [file microorganisms-07-00343-s001.zip › TableS1.docx]

| **Analytical constituents** | |
| --- | --- |
| Crude proteins | 18.00% |
| Crude cellulose | 4.3% |
| Crude fat | 7.0% |
| Minerals | 5.3% |
| Lysine | 1.62% |
| Methionine | 0.63% |
| Calcium | 0.61% |
| Sodium | 0.25% |
| Phosphorus | 0.55% |
| **Trace elements (mg/kg)** | |
| Iron | 101 |
| Iodine | 1.5 |
| Copper | 151 |
| Manganese | 120 |
| Zinc | 131 |
| **Vitamins (IU/kg)** | |
| A | 16 000 |
| D3 | 2000 |
| E | 150 |
| C | 100 |
