# Supplementary figures and images for: Microbiota Composition and Functional Profiling Throughout the Gastrointestinal Tract of Commercial Weaning Piglets
Source: Microorganisms. 2019 Sep 12;7(9):343. doi: 10.3390/microorganisms7090343 (PMC6780805; doi:10.3390/microorganisms7090343)

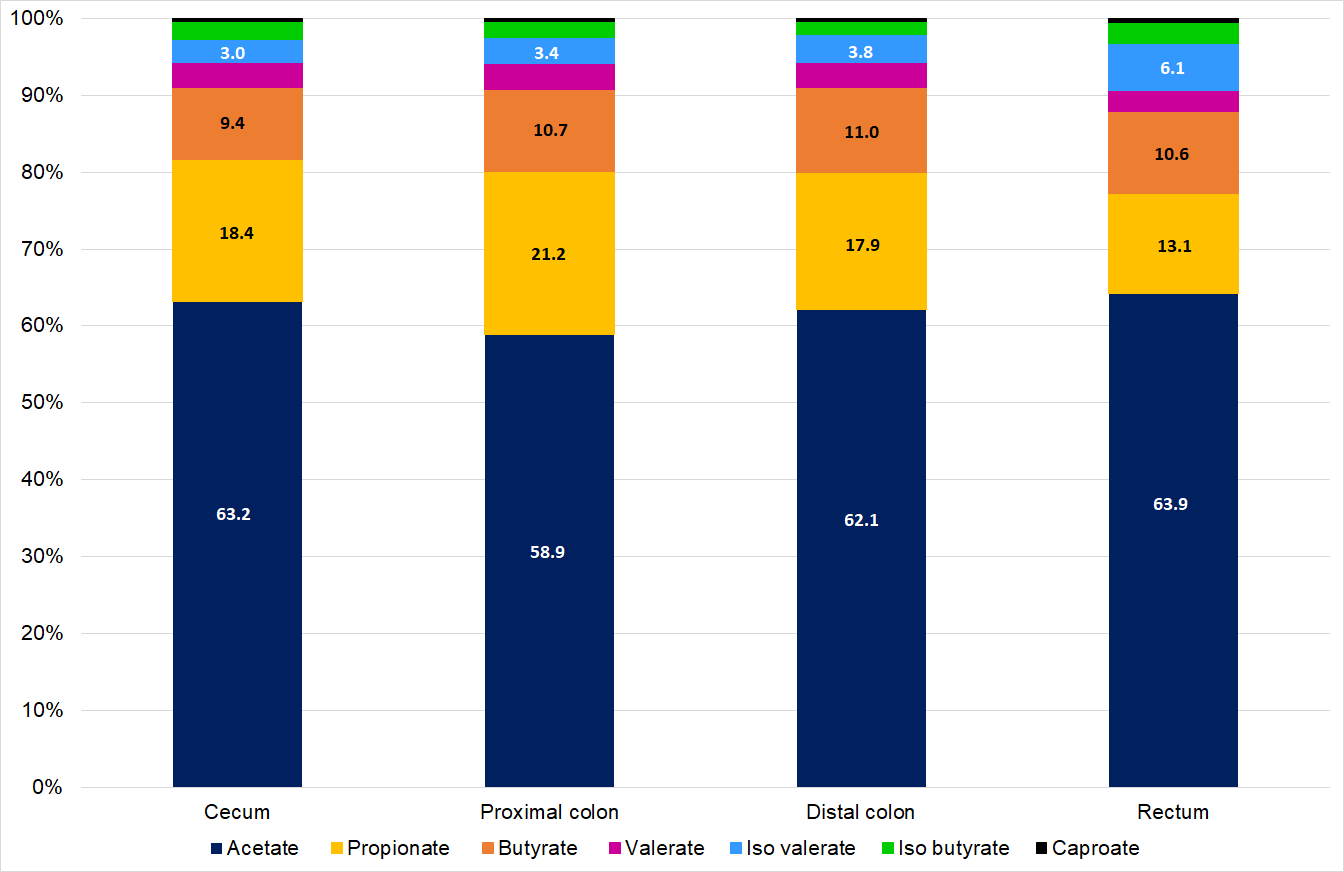

Supplement: Supplementary file 1 [file microorganisms-07-00343-s001.zip › FigureS2.png]

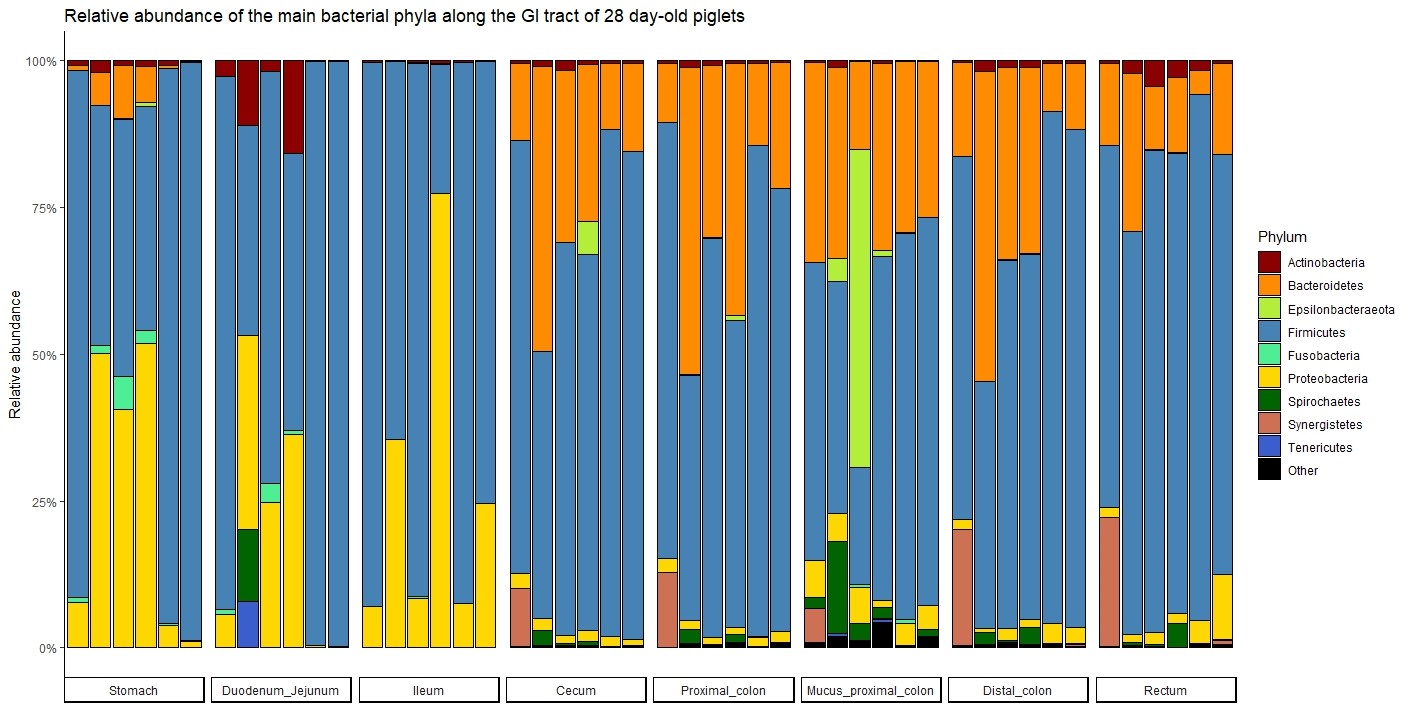

Supplement: Supplementary file 1 [file microorganisms-07-00343-s001.zip › FigureS3.jpg]

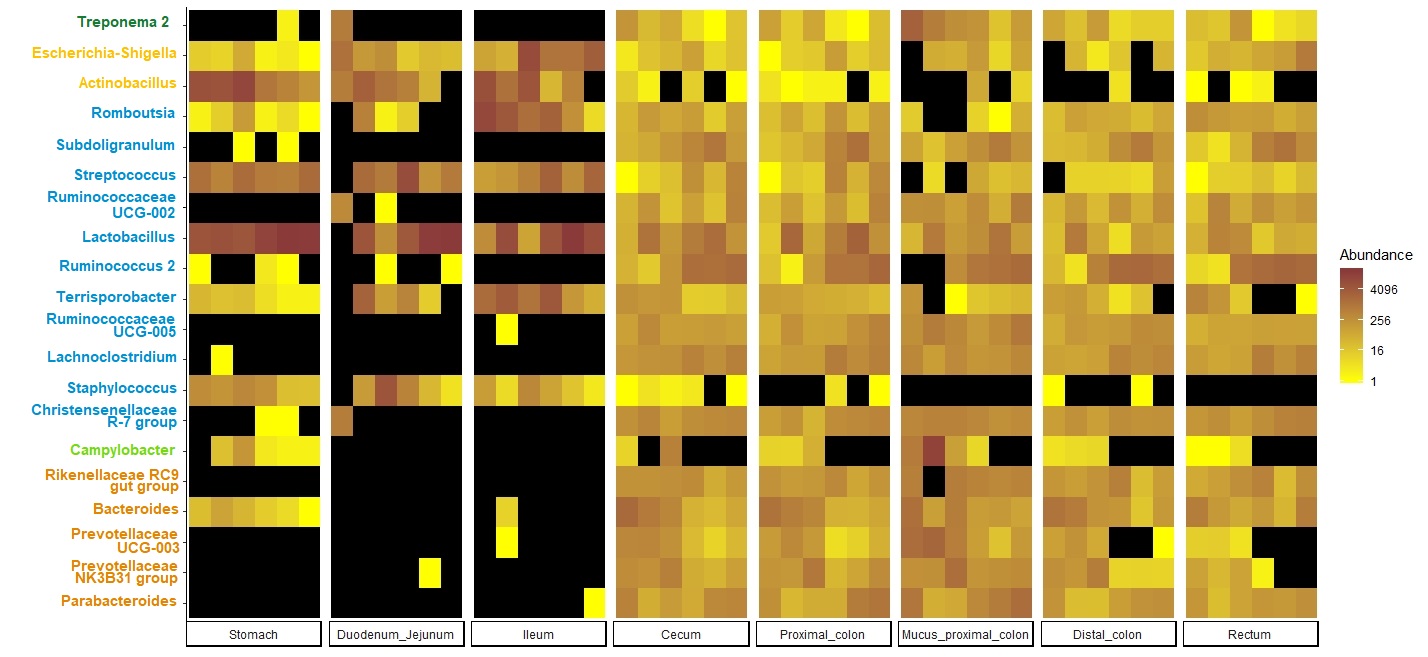

Supplement: Supplementary file 1 [file microorganisms-07-00343-s001.zip › FigureS4.jpg]

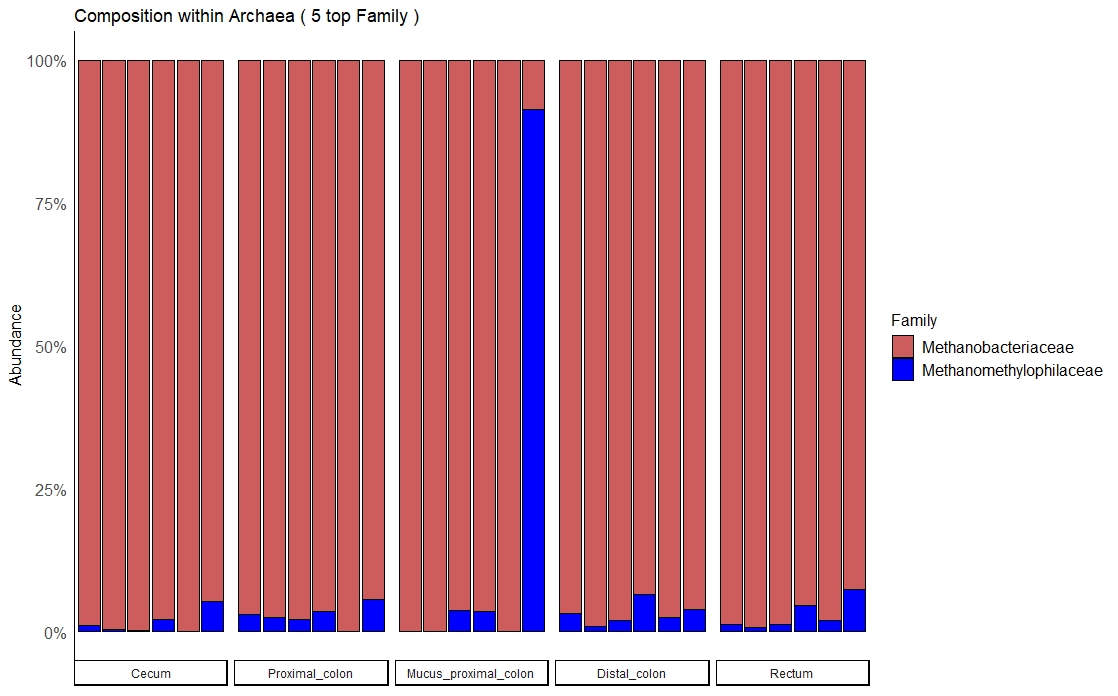

Supplement: Supplementary file 1 [file microorganisms-07-00343-s001.zip › FigureS5.jpeg]

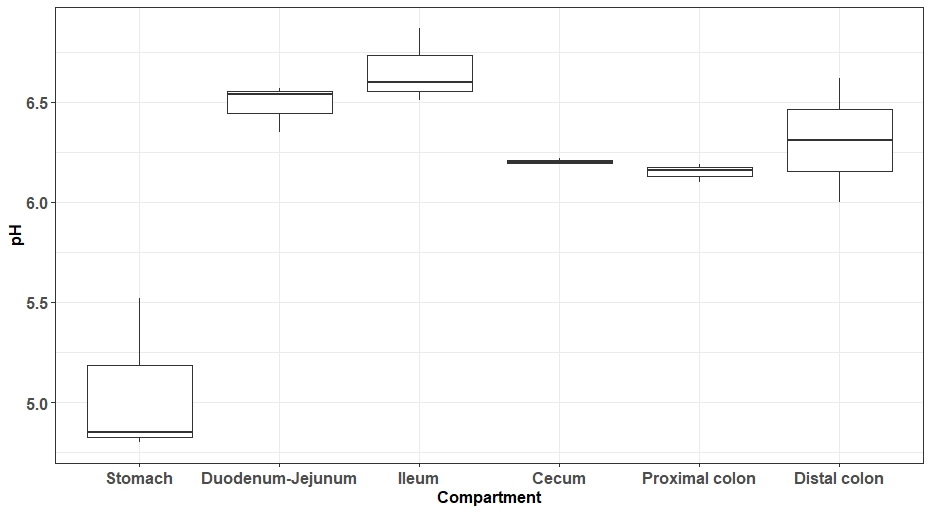

Supplement: Supplementary file 1 [file microorganisms-07-00343-s001.zip › FigureS1A.jpeg]

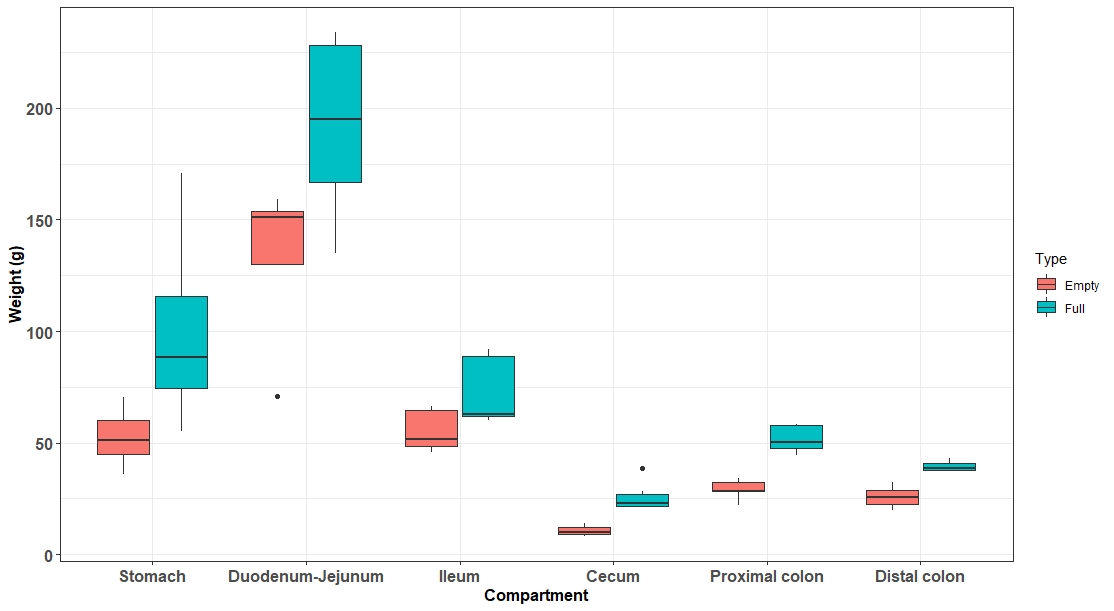

Supplement: Supplementary file 1 [file microorganisms-07-00343-s001.zip › FigureS1B.jpeg]
